# Aneurysm Characteristics Associated with the Rupture Risk of Intracranial Aneurysms: A Self-Controlled Study

**DOI:** 10.1371/journal.pone.0142330

**Published:** 2015-11-05

**Authors:** Huibin Kang, Wenjun Ji, Zenghui Qian, Youxiang Li, Chuhan Jiang, Zhongxue Wu, Xiaolong Wen, Wenjuan Xu, Aihua Liu

**Affiliations:** Department of Interventional Neuroradiology, Beijing Neurosurgical Institute, Beijing Tiantan Hospital, Capital Medical University, Beijing, China; Heinrich-Heine University, GERMANY

## Abstract

This study analyzed the rupture risk of intracranial aneurysms (IAs) according to aneurysm characteristics by comparing the differences between two aneurysms in different locations within the same patient. We utilized this self-controlled model to exclude potential interference from all demographic factors to study the risk factors related to IA rupture. A total of 103 patients were diagnosed with IAs between January 2011 and April 2015 and were enrolled in this study. All enrolled patients had two IAs. One IA (the case) was ruptured, and the other (the control) was unruptured. Aneurysm characteristics, including the presence of a daughter sac, the aneurysm neck, the parent artery diameter, the maximum aneurysm height, the maximum aneurysm width, the location, the aspect ratio (AR, maximum perpendicular height/average neck diameter), the size ratio (SR, maximum aneurysm height/average parent diameter) and the width/height ratio (WH ratio, maximum aneurysm width/maximum aneurysm height), were collected and analyzed to evaluate the rupture risks of the two IAs within each patient and to identify the independent risk factors associated with IA rupture. Multivariate, conditional, backward, stepwise logistic regression analysis was performed to identify the independent risk factors associated with IA rupture. The multivariate analysis identified the presence of a daughter sac (odds ratio [OR], 13.80; 95% confidence interval [CI], 1.65–115.87), a maximum aneurysm height ≥7 mm (OR, 4.80; 95% CI, 1.21–18.98), location on the posterior communicating artery (PCOM) or anterior communicating artery (ACOM; OR, 3.09; 95% CI, 1.34–7.11) and SR (OR, 2.13; 95% CI, 1.16–3.91) as factors that were significantly associated with IA rupture. The presence of a daughter sac, the maximum aneurysm height, PCOM or ACOM locations and SR (>1.5±0.7) of unruptured IAs were significantly associated with IA rupture.

## Introduction

Unruptured intracranial aneurysms (IAs) are found in 3–8% of the general population [1]. Although the annual rupture rate is fairly low, approximately 2% [2] of patients present with subarachnoid hemorrhage (SAH). Aneurysmal SAH remains associated with high morbidity and mortality, with mortality rates between 25 and 50% [3]. Whether predicting IA rupture is possible has been a controversial topic [4]. Many researchers have reported that hypertension increased the risk of aneurysm rupture [5–9]; however, other researchers have reported that hypertension was not a risk factor for aneurysm rupture [10–12]. Similarly, previous studies have reported that gender differences, smoking, and alcohol consumption were independent risk factors for aneurysm rupture [13, 14], while other studies have reported that these demographic variables were not risk factors for aneurysm rupture [9, 15, 16]. Many studies have reported that aneurysms with larger diameters (>5 mm or >7 mm) had a significantly greater rupture risk [6, 13, 17]. However, a study of 1256 cases of sporadic ruptured IAs in a single Chinese institution reported that ninety percent of ruptured IAs were less than 10 mm in size in patients with multiple IAs [18]. Other studies also had similar findings [19, 20]. When we studied aneurysmal risk factors (e.g., size and location), excluding the effects of demographic risk factors (e.g., age, sex, and hypertension) related to aneurysm rupture in a multivariate regression model was difficult because of differences among the individual patients. In patients with aneurysms of similar sizes and geometries, vessel-wall properties and flow characteristics could also vary substantially because of differences in patients’ demographic risk factors [21]. Thus, such confounding demographic risk factors may have led to statistical bias in previous studies. These discrepancies have fueled the search for new and better methods to study IA rupture. Several studies have reported using a case-control study model in patients with multiple IAs to identify the risk factors of IA rupture. In these models, the IAs that had ruptured composed the case group, and the other(s) that had not ruptured composed the control group; thus, each patient served as their own internal control [22, 23]. Based on previous studies, we conducted this 1:1 (self) case-control study model to study the natural risk factors of IA rupture.

## Methods

### Ethics Statement

The study protocol was approved by the Institutional Review Board of Beijing Tiantan Hospital. All of the patients provided written, informed consent to participate in the study, and the privacy of the patients was strictly protected.

### Study Population and Inclusion/Exclusion Criteria

Consecutive patients with newly diagnosed SAH (based on computed tomography [CT] scan images) and two aneurysms (one ruptured and one unruptured) were enrolled from January 2011 to April 2015. SAH was confirmed, and the ruptured aneurysm was identified by comparing CT images using digital subtraction angiography (DSA). All patients with clinically suspicious SAH underwent head CT scans without contrast. Patients with confirmed SAH immediately underwent DSA to determine whether an IA was the cause of the SAH. Ruptured IAs were systematically confirmed by two chief neurosurgeons (A Liu, C Jiang) who were blinded to the patients’ conditions. If a disagreement occurred, a third chief neurosurgeon (Y Li) was consulted. We excluded patients with only one IA or more than two IAs. Patients for whom the etiology of a ruptured aneurysm (based on CT scanning and DSA) could not be determined were excluded from our study (e.g., patients with two adjacent IAs in the same artery or two anatomically adjacent aneurysms).

#### Collection of Other Demographic Information

In this study, we collected demographic information for each patient, including age, gender, smoking status, alcohol use, and whether he or she had heart disease, hypercholesterolemia, and diabetes mellitus.

### Imaging

Detailed clinical records and imaging data (obtained via CT angiography, magnetic resonance angiography and DSA) were collected. All of the participating patients underwent conventional DSA imaging of the bilateral internal carotid arteries, bilateral external carotid arteries, and bilateral vertebral arteries. The patients also underwent 3D angiography, which allowed for the measurement and assessment of the arterial supplies and lesion locations.

### Definitions of the Parameters and Measurements

**Fig 1:**
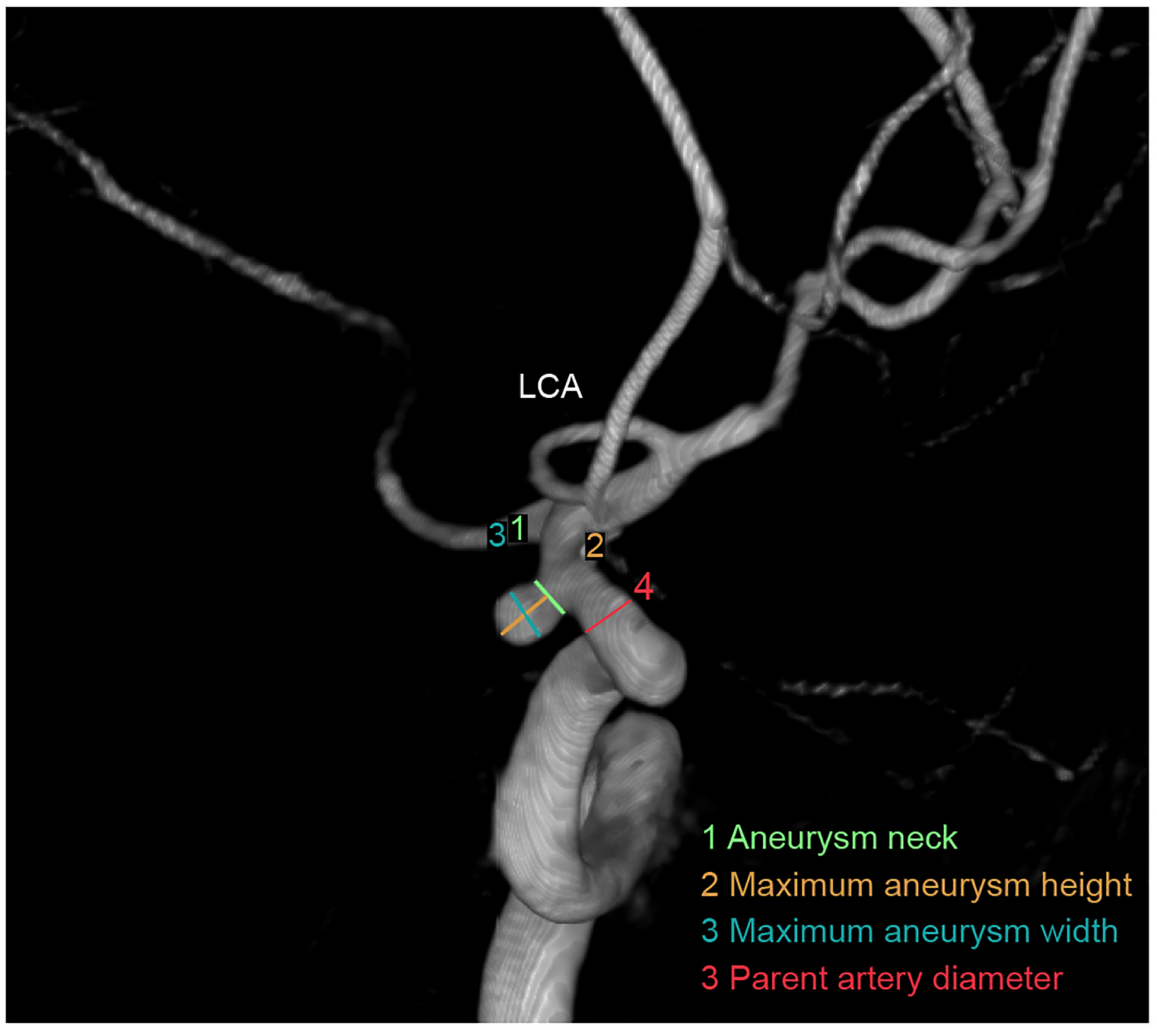
Aneurysm parameter definitions.

A daughter sac was defined as an irregular protrusion of the aneurysm wall [24]. The aneurysm neck diameter was calculated as twice the average distance from the neck centroid to the edge of the neck [25]. The parent artery diameter was obtained by averaging two representative vessel cross sections upstream of the aneurysm; each local diameter was calculated in the same manner as the neck diameter [25]. The maximum aneurysm height (aneurysm size) was measured as the maximum perpendicular distance between the dome and the neck plane [26]. The maximum aneurysm width was measured as the maximum horizontal length of the aneurysm. The aspect ratio (AR; maximum perpendicular/average neck diameter) was defined as the ratio of the maximum perpendicular height to the average neck diameter, and the average neck diameter was calculated as twice the average distance from the neck centroid to the edge of the neck [27, 28]. The size ratio (SR) was defined as the maximum aneurysm height/average of the parent diameter. The aneurysm-to-vessel SR incorporated the geometries of the IA and its parent vessel [25]. The maximum aneurysm width/maximum aneurysm height (WH ratio) was defined as the ratio of the maximum aneurysm width to the maximum perpendicular height (Fig 1).

We identified the sizes of the aneurysms and performed statistical analyses of the average sac measurements obtained by two readers. The aneurysmal measurements were made by two neuroradiologists (with an average of 10 years of practical academic experience) and by a clinical technologist (A Liu). Repeated measurements were made by C Jiang and a technician.

### Statistical Analyses

Statistical analyses were performed using Statistics Analysis System (SAS) software, version 9.3. The associations between the aneurysm characteristics and the rupture risk were analyzed. Two-sided values of *P*<0.05 were considered statistically significant. The distributions of the continuous variables and descriptive statistics (i.e., means, standard deviations, and 95% confidence intervals [CIs]) were generated. Paired *t*-tests were used to examine differences. Categorical variables were analyzed using paired Pearson’s χ^2^ tests. Variables that were not normally distributed were analyzed using the Mann-Whitney U test. Conditional, backward, stepwise multivariate logistic regression analysis was performed to identify the independent risk factors associated with IA rupture.

## Results

A total of 103 consecutive patients with 206 IAs, including 103 ruptured and 103 unruptured IAs, were included in this study. Of these patients, 28 were male, and 75 were female. The mean age was 56.5±11.5 years. The baseline demographic characteristics of the patients are shown in Table 1. The IAs were divided into a case group (ruptured) and a control group (unruptured) in accordance with the imaging findings. The baseline data of aneurysm characteristics and risk factors for the case and control groups are summarized in Table 2.

**Table 1:** Baseline demographic characteristics of the patients.

	NO. of Patients
Gender	
Male	28
Female	75
Age	56.5±11.5
Hypertension	
No	55
Yes	48
Heart disease	
No	97
Yes	6
Hypercholesterolemia	
No	92
Yes	11
Diabetes mellitus	
No	91
Yes	12
Smoking	
No	74
Yes	29
Alcohol use	
No	73
Yes	30

**Table 2:** Comparison of aneurysm characteristics between groups.

	Case (rupture)	Control (unruptured)	*P* Value
Daughter sac			0.006
Yes	14	89	
No	3	100	
Aneurysm neck	3.4±1.5	3.1±1.4	0.037
Parent artery diameter	3.9±1.0	3.6±0.9	0.422
Maximum aneurysm height			
Mean±SD (mm)	6.1±4.2	4.1±2.1	
Distribution			0.001
<7 mm	80	95	
≥7 mm	23	8	
Maximum aneurysm width			
Mean±SD (mm)	5.1±3.0	3.8±1.8	
Distribution			0.021
<7 mm	84	92	
≥7 mm	19	11	
Aneurysm location			0.003
PCOM and ACOM	41	60	
Other	62	43	
Aspect ratio	1.5±0.5	1.3±0.5	0.003
SR	1.5±0.7	1.1±0.7	<0.001
WH ratio	0.9±0.4	1.1±0.5	0.084

PCOM, internal carotid-posterior communicating artery; ACOM, anterior communicating artery; Other: MCA, middle cerebral artery; ICA, internal carotid artery; BA, basilar tip and basilar-superior cerebellar artery; VA, vertebral artery-posterior inferior cerebellar artery and vertebrobasilar junction; ACA, anterior cerebral artery.

### Univariate and Multivariate Analyses

The univariate analysis revealed that the presence of a daughter sac, a maximum aneurysm height ≥7 mm, location on the posterior communicating artery (PCOM) or anterior communicating artery (ACOM), the AR and the SR (1.5±0.7) were significantly associated with intracranial aneurysm rupture (P<0.05; Table 2). Then, a logistic regression model that employed a backward stepwise conditional method was conducted (Table 3). The adjusted ORs and P-values for the factors associated with IA rupture are shown in Table 3. Further conditional, backward, stepwise multivariate logistic regression analyses indicated that the presence of a daughter sac (OR, 13.80; 95% CI, 1.65–115.87), a maximum aneurysm height ≥7 mm (OR, 4.80; 95% CI, 1.21–18.98), location on the PCOM or ACOM (OR, 3.09; 95% CI, 1.34–7.11) and the SR (OR, 2.13; 95% CI, 1.16–3.91) were significantly associated with IA rupture (Table 3).

**Table 3:** Risk factors of aneurysm rupture according to conditional multivariate analyses.

	Hazard Ratio (95% CI)	*P* Value
Daughter sac	16.45 (1.88–143.94)	0.011
Maximum aneurysm height ≥7 mm	5.04 (1.21–20.99)	0.026
Location on PCOM and ACOM	3.09 (1.34–7.11)	0.008
SR	1.95 (1.11–3.42)	0.021

PCOM, internal carotid-posterior communicating artery; ACOM, anterior communicating artery; HR, hazard ratio.

## Discussion

In the present study, we utilized a self-controlled model that excluded all potential demographic rupture risk factors to identify risk factors related to IA rupture. The presence of a daughter sac, a maximum aneurysm height ≥7 mm, aneurysm location on the PCOM or ACOM, and the SR (>1.5±0.7) were associated with IA rupture.

A daughter sac is defined as an irregular protrusion of the IA wall [24]. Tominari et al. [24] studied 5651 Japanese patients with 6606 unruptured IAs. These authors found that the presence of a daughter sac was a predictor of the rupture risk, and the hazard ratio was as high as 1.48 for the presence of a daughter sac. Previous studies have suggested that aneurysm shape is an important risk factor for rupture, and this association is supported by both morphological and epidemiological studies [17, 26, 27]. In this study, the presence of a daughter sac increased the rupture risk by 16.45-fold after excluding all potential demographic rupture risk factors; this finding indicated that the presence of a daughter sac was associated with IA rupture.

Recently, American Heart Association/American Stroke Association (AHA/ASA) guidelines and other large studies on the management of patients with unruptured IAs have focused on the size and location of IAs [29–31]. Korja [32] conducted a study to identify IAs that have a high or low rupture risk. The author found that the presence of an unruptured IA of ≥7 mm was an independent risk factor for the lifetime occurrence of SAH and that the hazard ratio was as high as 4.02 for an IA ≥7 mm. The rupture risk for unruptured IAs <7 mm was minimal (<1% per year) [17, 33]. Small unruptured IAs had a low rupture risk [17, 27], and the rupture risk of large unruptured IAs (≥7 mm), though higher, remained relatively low [34]. Consistent with the findings of other large, prospective, multicenter studies [17, 30, 33], patients with IAs larger than ≥7 mm in diameter exhibited a significantly increased rupture risk. Other studies also reported that the average maximum size (≥7 mm) of the unruptured IAs was significantly smaller than the maximum size of ruptured IAs [4, 17, 35]. In contrast, a study of 1256 cases of sporadic ruptured IAs in a single Chinese institution reported that ninety percent of patients had ruptured IAs less than 10 mm in size [18]. Other studies also had similar findings [19, 20]. However, all of these studies have similar limitations. Although these studies performed multivariate analyses and found that size was an independent risk factor for the rupturing of unruptured IAs, these multivariate analyses were not able to fully adjust for all potentially confounding demographic factors. In the present study, we excluded all potential demographic rupture risk factors from our multivariate analysis and found that a maximum aneurysm height ≥7 mm was associated with a greater rupture risk compared with a maximum aneurysm height <7 mm. We found that the OR was 5.04 for a maximum aneurysm height ≥7 mm.

Previous studies have reported that IAs located in the ACOM [17, 35] or PCOM [17, 31] were significantly associated with the rupture risk. In our study, ACOM and PCOA locations were significantly associated with the rupture risk and increased the rupture risk by a factor of 3.09 after all potential demographic risk factors were excluded.

Previous studies have reported that a high AR was associated with IA rupture [28, 31], but not all studies showed that mean ARs were higher in ruptured IAs than in unruptured IAs [33–39]. Hoh and Backes showed that a high AR was associated with IA rupture in their case-control study model [22, 23]. In this 1:1 self-controlled study, the AR (1.5±0.5) was significantly associated with IA rupture in the univariate analysis but was not significantly associated with IA rupture in a logistic regression model after the exclusion of potential demographic risk factors. A possible reason for this finding is that the sample size is limited in this study; this limitation could have affected the result. Thus, further studies with larger patient series are needed.

The SR (maximum aneurysm height/average parent diameter) was a promising new morphological parameter for the assessment of IA rupture [25]. Dhar [25] found that the SR exhibited the strongest independent correlation with the rupture of an IA and that an unruptured group exhibited significantly smaller SRs than did a ruptured group [4]. The results of the present study agreed with this finding; IAs with larger SRs exhibited a greater rupture risk (OR, 1.95).

### Study Limitations

This study has several limitations. First, our study has a retrospective design and a relatively small sample size. The ruptured IAs were already known at the time of diagnosis. The number of patients with two aneurysms (one ruptured and the other unruptured) was relatively small, and 5 patients with no definite IAs corresponding with SAH via CT scanning and DSA were excluded. Further prospective studies with larger patient series are needed. Second, in discussing the factors (daughter sac, size ratio and aspect ratio) related with shape of ruptured IAs, we do not have proofs showing shape does not change by the event of rupture. We can easily imagine ruptured IAs can have irregular shape by the obstructed clots etc. Although Rahman, et al [40]showed there was minimal change in the size after the aneurysms rupture, there are no reports showing the shape does not change after the rupture. Hence, the minimal change in the size may affect the measurements of parameters related with the shape, possibly leading to bias. Third, aneurysm rupture could be visually verified during surgical clipping. However, surgical clipping and endovascular treatment occur in two separate departments in our center, and most patients with IAs underwent endovascular treatments. Therefore, we confirmed the ruptured aneurysms that caused the SAH within each patient by CT scanning and DSA; accordingly, some subjective bias could have been introduced. Fourth, the parent vessel vasospasm near the ruptured aneurysm affects the calculation of size ratios, possibly leading to bias.

## Conclusions

We concluded that the presence of a daughter sac, the maximum aneurysm height, a PCOM or ACOM location and the SR (>1.5±0.7) of an unruptured IA were associated with IA rupture. Moreover, the 1:1 (self) case-control study model of two IAs within the same patient was a simple and reliable model for studying the IA rupture risk.
